# A Method for Clustering and Cooperation in Wireless Multimedia Sensor Networks

**DOI:** 10.3390/s100403145

**Published:** 2010-03-31

**Authors:** Mohammad Alaei, Jose M. Barcelo-Ordinas

**Affiliations:** Computer Architecture Department, Universitat Politècnica de Catalunya (UPC), Barcelona, Spain; E-Mail: joseb@ac.upc.edu

**Keywords:** Wireless Multimedia Sensor Network (WMSN), clustering, field of view, cooperation, energy conservation, object detection, scheduling

## Abstract

Wireless multimedia sensor nodes sense areas that are uncorrelated to the areas covered by radio neighbouring sensors. Thus, node clustering for coordinating multimedia sensing and processing cannot be based on classical sensor clustering algorithms. This paper presents a clustering mechanism for Wireless Multimedia Sensor Networks (WMSNs) based on overlapped Field of View (FoV) areas. Overlapping FoVs in dense networks cause the wasting of power due to redundant area sensing. The main aim of the proposed clustering method is energy conservation and network lifetime prolongation. This objective is achieved through coordination of nodes belonging to the same cluster to perform assigned tasks in a cooperative manner avoiding redundant sensing or processing. A paradigm in this concept, a cooperative scheduling scheme for object detection, is presented based on the proposed clustering method.

## 1. Introduction

Wireless Sensor Networks (WSN) [1,2] are considered as autonomous and self-organized systems consisting of a large number of small, inexpensive, battery-powered communication devices deployed throughout a physical space. These networks are mainly used for gathering information related to the surrounding environment (e.g., temperature, humidity, light, *etc*.), and for transmission of the gathered data to a base station (*i.e.*, sink), for further processing. In recent times there has been increased interest in video surveillance and environment monitoring applications. Visual information may be captured from the environment using CMOS cameras embedded in wireless sensor nodes. Wireless Multimedia Sensor Networks (WMSN) [3,4], should be able to process in real-time, retrieve or fuse multimedia data.

Energy conservation and maximization of network lifetime are commonly recognized as a key challenge in the design and implementation of WSNs. One of the subjects that have been propounded for enhancement in efficiency of applications associated with WSNs is node clustering. Clustering in WSN pursues several objectives: (i) network scalability, (ii) energy conservation, (iii) network topology stabilization, (iv) routing overhead minimization, (v) optimized management strategies to prolong battery life and network lifetime and (vi) efficient data aggregation. Most of the time, distance from nodes to cluster-head or radio coverage (*i.e.*, neighbourhood) are the main criterions for node clustering in WSN [5].

Nevertheless, the sensing region of multimedia nodes is very different from ordinary nodes in WSNs. Each multimedia node has a Field of View (FoV) and can only capture images from the objects within that region. In traditional WSNs, the sensor nodes collect information about different phenomena around them from the area determined by the sensing range of the node. However, video cameras capture images of objects of a region that is not necessarily in the camera’s vicinity. The object covered by the camera can be distant from the camera and the captured images will depend on the relative positions and orientation of the cameras towards the observed object [6–8]. Therefore, because of non-coincidence between radio neighbourhood and sensed region by multimedia nodes, node clustering and coverage techniques in WSN do not satisfy WMSN requirements.

As a result of developments in low cost, low power, low resolution camera sensors, having a dense network consisting of multimedia sensors has become applicable. This kind of deployment has more performance than sparse networks of high power, high resolution cameras. However, overlapping FoVs in dense deployments yield wasting of power in the network because of redundant sensing of the area [7].

In this paper, we first propose an approach for multimedia node clustering that satisfies FoV constraints. In this approach, the overlapping area between FoV of multimedia nodes is the main criterion for node clustering in contrast to radio or distance neighbourhood. If the overlapped area between FoV of two nodes is relatively wide, they act similarly from the coverage point of view and thus we select them as members of a cluster. Thus, nodes belonging to the same cluster may not necessarily be neighbours. The main aim of this clustering method is energy conservation and network lifetime prolongation through the creation of potential of cooperation among nodes belonging to the same cluster and avoiding redundant sensing and processing. Applications may range from multiple-perspective monitoring and localization to collaborative object detection or distributed video coding. Second, we present a cooperative scheduling scheme for object detection as an example of an application which can be developed based on the proposed clustering method. The environment sensing task is divided in clusters of overlapping FoV areas. The members of each cluster cover a region with a high degree of overlapping; as an object can appear in one of these regions, the members of all clusters sense their domains concurrently in a collaborative manner. In each cluster, nodes are scheduled to sequentially wake up, capture an image and monitor the presence of the object applying an object detection procedure and finally go to sleep mode in an intermittent way. Each node belonging to a given cluster is involved in image capturing from its unique perspective in its own timing. In this way, members of the cluster participate in image capturing and object detection sequentially by time intervals.

The main contributions of this paper are:

- A node clustering algorithm based on overlapping camera FoVs. Finding the intersection polygons and in particular computing the overlapped areas is the key issue to establish clusters and determine cluster membership.
- Scheduling members of clusters in order to perform object detection in a planned manner.
- Energy conservation in scheduled cluster members, compared to energy consumption in ordinary un-clustered object detection and thus greater network lifetime.

The remainder of the paper is organized as follows: Section 2 discusses related work. Section 3 describes the way of finding and calculating overlapping polygons and then proceeds to explain the clustering algorithm. Test results of the clustering algorithm are shown by Section 4. In Section 5 a cooperative scheduling based on the proposed clustering method for object detection is presented. Furthermore, power conservation development and network lifetime prolongation through employing the cooperative scheduling method are discussed. Finally conclusions are derived in Section 6.

## 2. Related Work

Most of the work in WMSN is related to multimedia sensor hardware [3,4]. Soro *et al*., [6], analysed how coverage-based algorithms designed for scalar sensors do not behave well in a video sensor network in terms of environment monitoring. The reason is the mismatch between sensor location and sensing region (*i.e.*, FoV). In [8], the authors propose an optimal polynomial time algorithm for computing the worst-case breach coverage in FoV sensor networks, where “breach” is defined as the closest distance of the moving target to any sensor. The algorithm uses FoV-Voronoi graphs where only FoV shared areas participate in the construction of the Voronoi graph. The algorithm is used to inspect the probability that a random deployment results in an unobserved path between initial and final observed regions. The authors of [7] present an algorithm that enables self configurable sensor orientation calculation. The goal is finding the camera orientation that minimizes occlusion viewpoints and overlapping areas. All of them assume random camera deployments. The main justification for random deployments, [6–8], is to obtain sensing redundancy and avoid the negative effect of occlusions. Other approaches consider the well-known “art-gallery” problem when the objective is that all points in a 2D-plane are covered by at least one camera.

Clustering has been studied in great depth in the field of wireless scalar sensor networks. The work [5] surveys present clustering protocols and algorithms in this field and provides the main keys for designing such algorithms. Cluster formation and cluster-head selection manners are the key factors in clustering algorithms. Most of the clustering algorithms form clusters based on sensor neighbourhood (radio-coverage) or based on the distance from the cluster-head. The number of clusters and cluster-size are parameters that usually impact cluster formation procedures. Obrackza *et al*., [9], state the fact that directional FoV should be the key parameter to form clusters in WMSN and also highlight examples in which video sensor spatial-based collaboration provides robust object detection by cross-validating information.

On the other hand, some algebra and geometric calculations are related to our research. Works [10–13] present different methods for testing whether two triangles intersect each other. Their techniques solve the basic sets of linear equations associated with the problem and exploit the relations between these sets to distinguish the existing overlaps.

Object detection is known as one of the main tasks in WMSNs. Rahimi *et al.*, [14], have employed their low power and inexpensive camera device, Cyclops, for this aim. There, Cyclops periodically captures images from the outside world and is able to construct the stationary background using the moving average of the series of the images. It further constructs a model of the foreground based on the absolute difference of the instantaneous image and constructed background. In their case, Cyclops periodically sleeps for intervals of 15 seconds and executes the algorithm after each wake-up. Hierarchical network architectures with multiple tiers are designed modularly with assigned tasks for each tier. As in SensEye [15], object detection can be achieved with lightweight low-cost cameras in a first tier. Cyclops [14] is an example of a sensor in this tier. Each sensor node is duty-cycled and awakened periodically to capture an image and detect the presence of new objects independently of other nodes. Object detection is performed via simple frame differencing. Object recognition can be achieved with more computationally capable cameras in a second tier. Finally, object tracking involves PTZ (Pan-Tilt-Zoom) cameras in a third tier. Several wireless platforms and cameras may be found in the literature applying these concepts: CITRIC, Panoptes, Meerkats [16–19].

Xiao *et al.*, [20], study object detection in a dense wireless scalar sensor network and propose an energy efficient wake up scheme for scalar nodes sensing a circle of radius R_s_ in the vicinity of a sensor. The scheme does not satisfy multimedia sensor networks, because of the egregious difference in coverage between scalar and multimedia sensor networks. Our scheduling approach differs in two main contexts: applying camera FoV as main parameter to define a sensing area and using FoV-based clustering to minimize energy consumption in object detection applications.

Our approach goes more in the direction of Liu *et al*., [21], that propose a dynamic node collaboration scheme, FoV-based, for target tracking applications. This scheme randomly deploys dynamically clusters and then applies sequential Montecarlo techniques to estimate target locations cooperatively. Here, we consider simpler methods to create clusters based on overlapping areas, while Liu *et al*. create clusters based on those cameras that can detect a specific target with high probability.

## 3. Node Clustering for WMSNs

**Figure 1.**
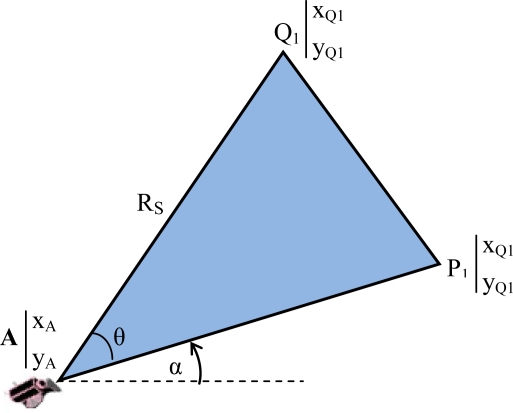
FoV of a multimedia sensor.

Multimedia sensors, such as cameras, are multidimensional sensors that can capture a directional view. We assume wireless sensor nodes with fixed lenses providing a θ angle FoV, densely deployed in a random manner. The assumption of fixed lenses is based on the current WMSN platforms. Almost all of them (SensEye, MicrelEye, CITRIC, Panoptes, Meerkats [15–19]) have fixed lenses and only high powered PTZ cameras have movement capabilities. We assume that sensors are aware of their position. We consider a monitor area with N wireless multimedia sensors, represented by the set S = {S_1_,S_2_,...,S_N_} randomly deployed. Each sensor node is equipped to learn its location coordinates and orientation information via any lightweight localization technique for wireless sensor networks. It is not the purpose of this paper to define mechanisms to find this location. Without loss of generality, let us assume that nodes in the set S belong to a single-tier network or the same tier of a multitier architecture. The following definitions will be used throughout the paper:

▪ *Field of View* (*FoV*): refers to the directional view (see Figure 1) of a multimedia sensor and it is assumed to be an isosceles triangle (two-dimensional approximation) with vertex angle θ, length of congruent sides R_s_ (sensing range) of the sensor and orientation α. The sensor is located at point A (x_A_,y_A_).
▪ *Cluster* (*C_j_*, *j = 1,…,M*): consists of a subset of multimedia nodes with high overlapping FoV areas. The size of the overlapping area between FoVs of two nodes determines whether they can be in the same cluster.
▪ *Overlapping scale* (*γ*): defines the minimum percentage of node’s FoV area that is required to be overlapped for membership in a cluster.

### 3.1. Overlapping Areas between FoV of Multimedia Nodes

**Figure 2.**
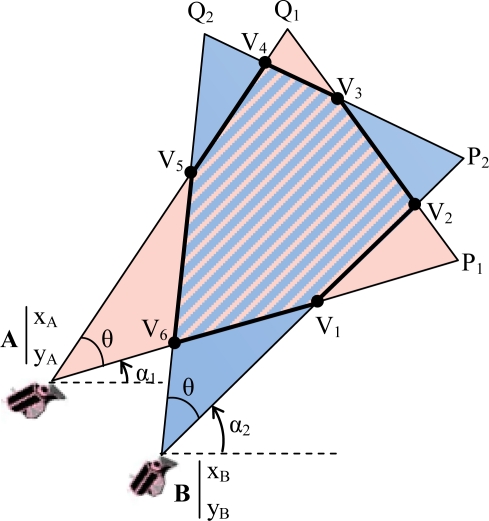
An example of the intersection polygon of two FoVs, (V_1_V_2_…V_6_).

It is obvious that there is no overlap between FoV of two nodes if the Euclidean distance between them is more than 2R_S_. Otherwise, it is possible to have overlapped regions between their FoV depending on the orientation angles α. For calculating the FoV overlapping area of two nodes, we first survey the intersection of triangles that are representatives of their FoVs. Second, if they intersect each other, we find the intersection polygon and at last, compute the area of the polygon. An example of the intersection polygon of two FoV belonging to nodes A = (x_A_,y_A_) with orientation *α*_1_ and B = (x_B_,y_B_) with orientation α_2_ is illustrated in Figure 2.

For this purpose, in the first step, we define the equations of the sides of each triangle using the vertex coordinates of each triangle. The coordinates of the main vertex A (Figure 1) are known according to the location of the node in space. The coordinates of vertex P_1_ and Q_1_ are:

(1) $$x_{P_{1}}=x_{A}+R_{S}\cdot \text{cos}(\alpha )$$

(2) $$y_{P_{1}}=y_{A}+R_{S}\cdot \text{sin}\;(\alpha )$$

(3) $$x_{Q_{1}}=x_{A}+R_{S}\cdot \text{cos}\;((\alpha +\theta )\text{mod}\;2\pi )$$

(4) $$y_{Q_{1}}=y_{A}+R_{S}\cdot \text{sin}\;((\alpha +\theta )\text{mod}\;2\pi )$$

We can determine the equation of a line from the coordinates of two points of the line or from the coordinates of one point and the gradient of the line. Thus, using the coordinates of A, P_1_ and Q_1_, we can determine the equations of the three sides of the FoV triangle:

(5) $$\bar{P_{1}Q_{1}}:y-y_{P_{1}}=\frac{y_{Q_{1}}-y_{P_{1}}}{x_{Q_{1}}-x_{P_{1}}}\cdot \left(x-x_{P_{1}}\right)$$

(6) $$\bar{{\text{AP}}_{1}}:y-y_{A}=\left(x-x_{A}\right)\cdot \text{tan}\;(\alpha )$$

(7) $$\bar{{\text{AQ}}_{1}}:y-y_{A}=\left(x-x_{A}\right)\cdot \text{tan}\;((\alpha +\theta )\text{mod}\;2\pi )$$

**Figure 3.**
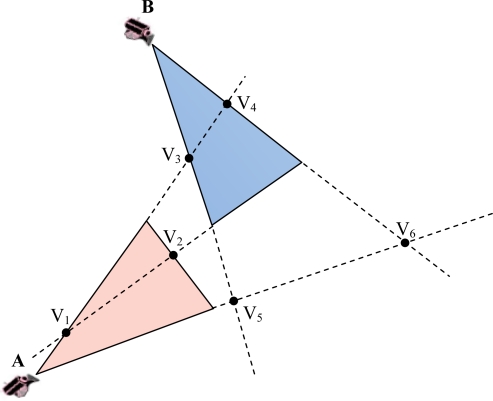
Non-acceptable intersection points, each two anti-parallel lines have an intersection point but the point have to satisfy Equation (8) to be a vertex of the intersection polygon.

In the second step, we calculate the intersection of each side of each triangle to all sides (*i.e.*, the perimeter) of the other triangle. An intersection point V of two lines representing two sides of the FoVs will be a vertex of the overlapped polygon if it lies among the vertices associated with those two sides. As illustrated in Figure 2, the line representing AP_1_ of the FoV of node A intersects the line that represents BP_2_ of the FoV of node B in point V_1_. V_1_ can be considered as a vertex of the intersection polygon because V_1_ is located between A and P_1_ and also between B and P_2_. The desired condition (C_ACCEPT_) for an intersection point V to be an acceptable vertex of the polygon is stated in Equation (8). This subject is noticeable because any two anti-parallel lines obviously have an intersection point in a two dimensional space. On the other hand, each side of a FoV is a segment of a line. Figure 3 shows examples of non-acceptable intersection points:

(8) $$C_{\text{ACCEPT}}=\left(\text{Min}\;\left(x_{A},x_{P_{1}}\right)\leq x_{V}\leq \text{Max}\left(x_{A},x_{P_{1}}\right)\right)\wedge \left(\text{Min}\;\left(x_{B},x_{P_{2}}\right)\leq x_{V}\leq \text{Max}\left(x_{B},x_{P_{2}}\right)\right)$$

**Figure 4.**
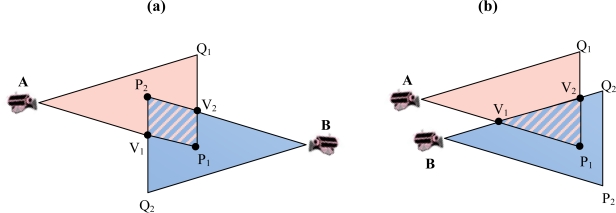
Examples of side intersection in only one point, for instance AP_1_ intersect the perimeter of the other triangle in only V_1_ in both (a) and (b).

The intersection of each side of one triangle with all sides of another triangle consists of at most two points. Figure 2 shows the case in which each side of each triangle intersects the perimeter of the other one in exactly two points V_i_ and V_j_, becoming the segment V_i_V_j_ one of the sides of the intersection polygon. However, there are other situations in which the intersection of one side of a triangle with the perimeter of another triangle occurs only at one point, resulting in that one of the vertices associated with that side lies within the second triangle and will become one of the vertices of the polygon (Figure 4a,b).

In the third step, a decomposition approach is used for calculating the area of the overlapped polygon in a 2D-plane. Let a polygon (W) be defined by its ordered vertices V_i_ = (x_i_,y_i_) for i = 0,...,n with V_n_ = V_0_. Also, let P be a reference point in the 2D-plane. For each edge V_i_V_i+1_ of the polygon W, form the triangle Δ_i_ = PV_i_V_i+1_. Then, the area of the polygon W is equal to the sum of the signed areas of all the triangles Δ_i_ for I = 0,...,n – 1:

(9) $$A(W)=\sum_{i=0}^{n-1}A\left(\Delta_{i}\right)\;\text{where}\;\Delta_{i}={\text{PV}}_{i}V_{i+1}$$

A(Δ_i_) refers to the area of triangle Δ_i_. Notice that, for a counter-clockwise oriented polygon, when the point P is on the left side of an edge V_i_V_i+1_, the area of Δ_i_ is positive; whereas, when P is on the right side of an edge V_i_V_i+1_, the area Δ_i_ is negative. If the polygon is oriented clockwise, then the signs are reversed. This computation gives a signed area for a polygon and similar to the signed area of a triangle, is positive when the vertices are oriented counter-clockwise around the polygon, and negative when oriented clockwise. We refer to [22] for a detailed description of the algorithm for calculating the area of a 2D polygon.

### 3.2. Cluster Formation and Cluster Membership

Now, let us consider the set S = {S_1_,S_2_,...,S_N_} of wireless multimedia nodes belonging to the same tier of a network randomly deployed. The cluster formation algorithm is executed in a centralized manner by the sink after deploying the network. The main reasons in choosing a central architecture are the following: (i) for a distributed architecture, each node should notify to the rest of the nodes about its location A_i_ and its orientation α_i_ (i = 1,…,N). In a centralized architecture the nodes should notify to the sink their location and orientation. Note that this notification can be done using any energy efficient sensor routing protocol and only is necessary at bootstrap phase. All phases of the clustering algorithm are executed only one time, right after node deployment. (ii) In many WSN applications, the sink has ample resources (storage, power supply, communication and computation) availability and capacity which make it suitable to play such a role. (iii) Collecting information by a sink node is more power efficient compared to spreading this information to each and every other node within the network. (iv) Having the global view of the network at the sink node facilitates provision algorithms for closer-to-optimal cluster determination; the global knowledge can be updated at the sink when new nodes are added or some nodes die. Such maintenance tasks can be regarded as a normal routine for the sink. (v) Finally, using a centralized scheme can relieve processing load from the sensors in the field and help in extending the overall network lifetime by reducing energy consumption at individual nodes. The following phases are performed to establish and form clusters:

▪ *Bootstrap*: At node bootstrap, each sensor {S_i_, i = 1,…,N} transmits its position (x_i_,y_i_) and orientation α_i_ to the sink. To accomplish this step any efficient sensor routing algorithm can be used. Thus, the clustering algorithm is not bound to how the sink receives this information. If there is an un-connected node in the network, it cannot announce itself and thus will not be considered in the algorithm.
▪ *Cluster Formation*: (i) Initially, the sink creates an empty cluster associated with an un-clustered multimedia node of S. Thus, that node will be clustered as the first member (*i.e.*, cluster-head) of the established cluster. (ii) Then, the sink finds the qualified un-clustered nodes for joining to that first member by computing the area of overlapped polygons of their FoV. From position and orientation of nodes, the sink computes the overlapped polygon area (D_ij_) between each un-clustered multimedia node and the first member of the established cluster as discussed in Section 3.1. If the computed overlapped area is equal or greater than the area determined by the overlapping scale (γ), the un-clustered node will be clustered as a member of the established cluster. (iii) When no more nodes can be added to the cluster, the sink takes a new un-clustered node, begins a new cluster and goes to step (ii). Table 1 indicates the formation procedure.
▪ *Membership notification*: we assume that the sink uses any energy-efficient sensor routing algorithm to notify to each first-member of every cluster about its cluster-ID and what are the members of the cluster. Then, each first-member sends a packet to the members of his cluster notifying them about the cluster which they belong to.

The algorithm is executed by the sink once upon deployment and thus all nodes will become clustered. If a node joins to the network hereinafter, it has to send its position and orientation to the sink for announcing itself as a new node. The sink computes the FoV of the new node and finds the first cluster that can accept it as a new member. For this purpose, the sink computes the overlapping regions between FoV of the new node and the first-member of each cluster and checks whether he is satisfying the cluster membership test. Then, the sink sends a message to the first-member in order that this node re-organizes the cluster with the new member. Depending on the application, this notification may suppose a new reconfiguration in the monitoring task (*i.e.*, a new duty-cycle period). On the other hand, each node periodically sends a Hello message to the first-member notifying that he is alive. When a node dies, the first-member will notify the rest of the members about the new cluster set and will reconfigure any parameter related to the cluster. The first-member also periodically notifies to his cluster members about his availability. If a first-member dies, the cluster members will notify to the sink their availability to belong to another cluster or to create a new cluster. Note that the beaconing among cluster members implies low overhead since cluster sizes have few nodes and hello periods can be on the order of duty-cycle sensing periods.

### 3.3. Cooperative Monitoring in Clusters

Let us see the potential of cooperative node monitoring in clusters in terms of sensor area coverage. We define the Maximum Cluster Coverage Domain (MCCD) parameter for a cluster as the maximum monitoring area which is covered by that cluster. Since each cluster is established considering an overlapping scale equal to γ, the MCCD can be computed as follows (C_size_ is the size of the cluster):

(10) $$\text{MCCD}=\gamma \cdot A_{\text{FoV}}+(1-\gamma )\cdot A_{\text{FoV}}\cdot C_{\text{size}}=\left(C_{\text{size}}-\gamma \cdot \left(C_{\text{size}}-1\right)\right)\cdot A_{\text{FoV}}=\beta \cdot A_{\text{Fov}}$$

where:

(11) $$1\leq \beta =C_{\text{size}}-\gamma \cdot \left(C_{\text{size}}-1\right)$$

The effective cluster covering domain can be inferior to the MCCD calculated by (10) since some nodes can overlap more than the region determined by γ. Since MCCD gives us an upper bound on the area covered by the cluster, using MCCD will allow us worst-case dimensioning. Factor β represents the increment of area that the cluster senses with respect to an individual sensor. When each node of a cluster obtains an image from its FoV, a part of the related MCCD with a ratio at least equal to 1/β respect to the MCCD is captured whereas this part includes overlapped areas of other nodes in the cluster. Sensing the environment by each member delivers information not only from the FoV of the active node but also from some overlapped parts of other nodes in the same cluster: at least γ·A_FoV_ of the area is common to the first-member and more than 1/β of the MCCD is monitored. For example, in a cluster consisting of just 2 members, assuming an overlapping scale of γ = 0.5, the MCCD is 1.5·A_FoV_. Thus, when each of the two members of the cluster is activated and monitors the environment, an area of one FoV is captured that is at least 2/3 of the whole MCCD of the cluster.

Consequently, scheduling and coordination among members in order to sense the field in a collaborative manner may yield a gain in energy saving and performance efficiency even with a low number of members in the cluster.

## 4. Cluster Formation Algorithm Evaluation

All sensor nodes have been configured with a FoV vertex angle of θ = 60° and R_S_ of 20 m. A sensing field spanning an area of 120 m × 120 m has been used. Sensor densities were varied to study the cluster formation from sparse to dense random deployments. Figures illustrate the average results of 50 independent running tests whereas each test corresponds to a different random deployment. Once a random deployment is defined, cluster formation is obtained from node location, angle of orientation and FoVs of nodes, using the described algorithm whose complexity is O(N·logN). Furthermore, as it was mentioned before, each node sends a packet to the sink in the bootstrap phase, then the sink notifies each first member via one packet his membership set for that cluster (phase 3) and then the first-members notify cluster nodes about their cluster membership and any related parameter. Thus, the average overhead of the algorithm is forwarding N packets from the nodes to the sink and forwarding N_C_ packets from the sink to first-members and forwarding N_C_·(μ_Csize_ – 1) packets from first-members to cluster nodes; where N is the number of nodes, N_C_ is the average number of clusters and μ_Csize_ is the average cluster size. So the total overhead will be: N + N_C_ + N_C_·(μ_Csize_ – 1) packets. The maintenance overhead is N_C_·(μ_Csize_ – 1) beacons every keep-alive period, where the keep-alive period can be a multiple of the sensing duty-cycle period.

### 4.1. Number of Clusters and Average Cluster-Size

**Figure 5.**
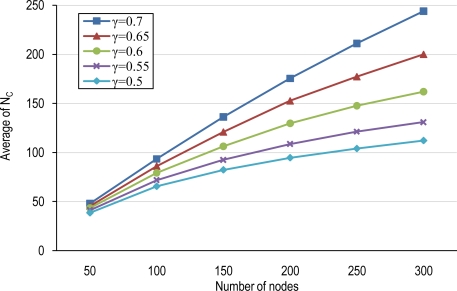
Average number of clusters (N_C_).

**Figure 6.**
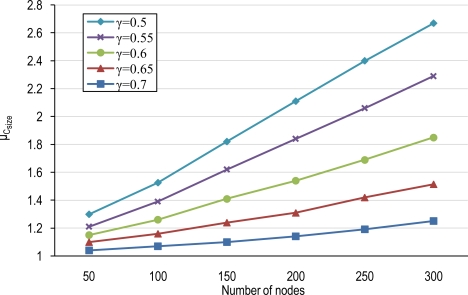
Average cluster-size (μ_Csize_) for different number of nodes and overlapping scales.

The average number of clusters, N_C_, and the average cluster-size (μ_Csize_) in a tier/network for different node densities with several overlapping scales are shown in Figures 5 and 6. Increasing the node density does not only cause an increment in the number of clusters but also yields more overlapping areas among FoVs and thus raises the cluster-size. However, the overlapping scale (γ) also impacts in the cluster membership selection process. The overlapping scale determines the minimum region that is required to be overlapped between the FoV of each node belonging to a given cluster and the FoV of the first member of that cluster. So, γ determines the minimum intersection part of FoV of each member with the first member of an established cluster. Lower overlapping scales obligate less overlapping areas for cluster membership and increase the domain covered by a given cluster since more nodes will be conforming to the membership rule. Increasing the overlapping scale restricts node membership because of higher required overlapping areas between FoVs of nodes. Thus, higher overlapping scales result in lower cluster-sizes, less MCCD and thus higher number of clusters.

Sparse networks have low average cluster-size, μ_Csize_, because sparse deployments result in low overlapping areas. Moreover, high values of γ also will produce low μ_Csize_. The result will be lower potential for node coordination. On the other hand, dense wireless multimedia sensor networks can particularly benefit from higher cluster sizes and thus more potential for node coordination.

**Figure 7.**
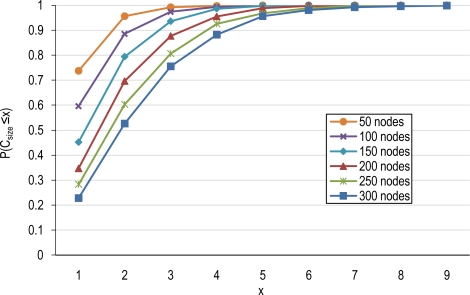
The cluster size cumulative distribution function as a function of node densities (γ = 0.5).

Finally, Figure 7 shows the cumulative probability function for the cluster-size in the network for different node densities assuming an overlapping scale of γ = 0.5. For example, in a network consisting of 250 nodes, 28% of clusters have a single member which does not have enough overlapping with others to satisfy the overlapping scale, 32% of clusters have a cluster size of 2, 21% of 3, 12% of 4 and 7% of them consisting of more than four members.

### 4.2. Covering Domain

**Figure 8.**
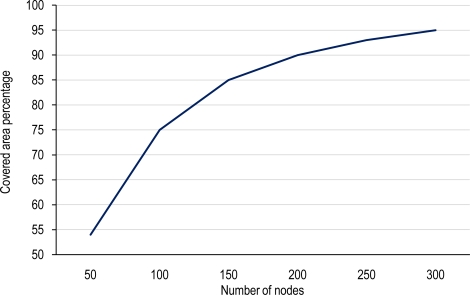
Percentage of the covered area with respect to the whole deployment area.

Figure 8 illustrates the percentage of area that is covered by the random deployment in terms of node density. As it is shown in the figure, for covering 95% of the area, a dense deployment of 300 nodes is required. As the figure shows, the rate of increment of the covered area for low node densities is faster than for high node densities. This indicates that after a new node is added in a dense deployment, low new coverage area is obtained.

For example, the first 100 nodes cover 75% of the field, but the next 100 nodes will only cover 15% of new area. The conclusion is that dense networks are able to cover high areas at the cost of high overlapping and sensing redundancy, but this overlapping can be used for improving reliability if nodes belonging to the same cluster work in a coordinated manner. Furthermore, the existence of obstacles produces a reduction of the sensing area because of FoV occlusion effect, [7]. So, employing dense networks of low-cost, low-resolution and low-power multimedia sensor nodes instead of sparse networks of high-power, high-resolution sensors (e.g., PTZ) will be more beneficial.

Applications that are interested in multiple views will also benefit from this situation, since there will be several nodes monitoring the same area from several perspectives. Applications that are interested in detecting objects and are not interested in having an instantaneous multiple-view of the object may benefit from collaborative node processing in terms of energy savings. For the first set of applications clustering of nodes may serves as an indicator of triggering simultaneous multi-perspective pictures. For the second set of applications, clustering may serve as a baseline framework for collaborative node scheduling avoiding redundant sensing and processing and thus increasing network lifetime. Other applications that are interested in correlated data (e.g., Distributed Video Coding, DVC) may use clustering in order to exploit multi-view correlations to build joint encoders [24].

## 5. Cooperative Scheduling for Object Detection in Cluster-Based WMSN

For object detection applications, sensor nodes are usually programmed to work in one of these two ways: (i) lower tier nodes (e.g., scalar sensors) detect movement and awake the camera sensors. (ii) The camera nodes sense the environment periodically.

In the first case, scalar nodes detecting movement have to be aware of which camera nodes have to be awakened. Awakening all nodes surrounding the scalar sensor does not assure detecting the object; camera sensors with their FoV targeting the object are not necessarily radio neighbours or even may not be in the surroundings of the scalar sensor. Knowing the area sensed by tier of cameras is not scalable, since the scalar sensor should keep information from all cameras that sense large areas covered by the scalar sensor. Finally, a possible solution is sending the information towards a sink that takes the responsibility of awaking nodes in the tier of cameras. However, these solutions do not seem good in terms of performance parameters such as delay and energy conservation.

**Figure 9.**
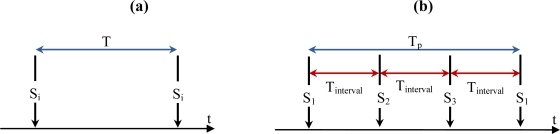
(a) Period of awakening a given node in the un-cooperative scheduling. (b) Scheduling for a cluster consisting of three members (S_1_, S_2_, S_3_).

In the second case, cameras should perform duty-cycled monitoring over the area that they sense. That means that every T seconds all sensors in the monitored area will awake and will perform object detection, see Figure 9.a. This is the situation for a planned network in which every sensor is placed in such a position that there is no overlapping among sensors. Nevertheless, this duty-cycle scheduling will produce high power consumption in those situations in which there are overlapping sensors, since camera nodes with overlapping areas do not cooperate to sense the area and thus they redundantly monitor the area.

In this section, we propose a cooperative mechanism based on the proposed clustering method that coordinates nodes belonging to the same cluster to work in a collaborative manner to detect objects. The objective of this mechanism is to increase power conservation by avoiding similar sensing and redundant processing at the same time. Also, collaborative sensing by nodes that have FoVs intersecting each other yields to more reliability: cluster members will monitor the region sequentially and if a moving object is not detected in one image capturing, it will be in the vicinal FoVs at the next capturing times. Thus, the other members in the same cluster may detect the object.

Let us divide the environment in domains covered by clusters of nodes (MCCD, Section 3.3) with certain degree of overlapping between members. All clusters concurrently sense their domains: in each cluster, members are awakened sequentially in an intermittent manner by the first member with a time interval (*i.e.*, T_interval_ is the time between awakening two consecutive members) related to the cluster-size and the scale of overlapping, see Figure 9(b). In this way, each node of a given cluster periodically participates in capturing an image from its unique perspective and surveying the presence of an object and finally sleeps again with a cluster-based period called T_p_. Formulas for these periods are derived in Section 5.1. Table 2 gives the pseudo-code for this algorithm.

### 5.1. Cluster-based T_P_ and T_interval_ Computation

Let us consider as baseline mechanism a non-collaborative duty-cycled scheme in which every node awakes with an interval period of time T and senses the area (*i.e.*, takes a picture and performs object detection) as tier 1 in [15]. The objective of the proposed collaborative mechanism is to produce a cluster-based duty-cycled scheduler in which: (i) Each node is awakened and senses the area with a reliable period of T_p_ > T taking advantage of the overlapping among nodes in the cluster, thus, saving energy and increasing network lifetime. Each cluster will have its own T_P_ interval, determined according to the cluster-size and the overlapping scale. (ii) During the sleeping period of each member of a given cluster, other nodes belonging to the cluster are awakened with intervals of T_interval_ < T (that is equal to: T_p_/C_size_) in a sequential manner.

The area sensed by each cluster is related to the MCCD area. In order to compute T_p_ we will consider the MCCD area. By awaking each member of a given cluster, in average, a part of the related MCCD with a ratio equal to 1/β is captured (Equation (11)). Note that the MCCD is an area of β.A_FoV_ and is sensed by C_size_ overlapping members, thus sensing the environment by each node delivers information not only from the FoV of the awakened node but also from some overlapped parts of the FoV of other nodes in the same cluster. Then, we may define the node interval duty-cycle period as:

(12) $$T_{P}=T\cdot \frac{C_{\text{size}}}{\beta }=T\cdot \frac{C_{\text{size}}}{C_{\text{size}}-\gamma \cdot \left(C_{\text{size}}-1\right)}$$

Note that the T_P_ is proprietary for each cluster in terms of its cluster-size and overlapping scale. As it was mentioned before, the MCCD calculated by Equation (10) is the maximum covering domain of a cluster while the effective cluster covering domain may be less than MCCD since some members of a given cluster may overlap more than the region determined by γ. Consequently, a given cluster can cover an area less than β·A_FoV_. Thus, using β gives us the lowest interval T_p_ and thus the most reliable one since lower values of β would increase the interval T_P_. On the other hand, members of a cluster are awakened sequentially to sense their environment in an intermittent way with time intervals equal to T_interval_:

(13) $$T_{\text{interval}}=\frac{T_{p}}{C_{\text{size}}}=\frac{T}{C_{\text{size}}-\gamma \cdot \left(C_{\text{size}}-1\right)}\leq T$$

Let us consider Figure 9(b) and for example a cluster with three members, C = {S_1_, S_2_, S_3_}, cluster-head S_1_ and γ = 0.5. Every node will be awakened every T_P_ = 1.5·T seconds and the area will be monitored every T_interval_ = 0.5·T seconds. As can be observed, every sensor is awakened with a period higher than the non-collaborative scheme but the area is monitored more times. Then, the area duty-cycled frequency is increased while the sensor duty-cycled frequency is reduced.

**Figure 10.**
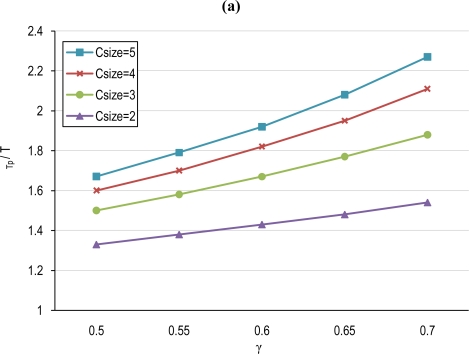

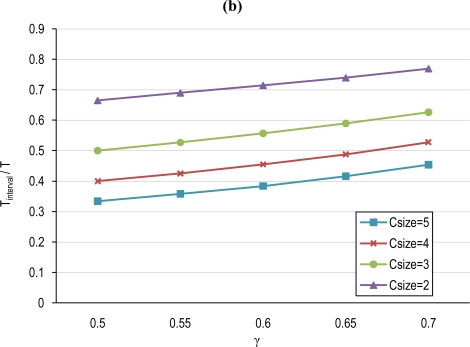
(a) Sensor node duty-cycle period T_p_/T, (b) Cluster duty-cycle period T_interval_/T.

Figure 10a,b shows the evolution of T_p_ and T_interval_ as a function of γ for several C_size_. Both parameters are normalized by T. We first have to notice that for a overlapping scale factor γ = 1, T_p_ = T, while for γ < 1, T ≤ T_p_ ≤ T/(1 – γ). Then the duty-cycle frequency at which a specific node is awakened is decreased by a factor that at least is (1 – γ) times the frequency of the non-collaborative scheme. On the other hand some sensor of the cluster will be on duty every T_interval_ seconds. Note that T_interval_ will be lower than T and will be smaller as C_size_ increases. This means that the area is monitored more frequently although every specific sensor monitors with less frequency. The reason is justified in how clusters are formed. Any sensor of the cluster overlaps with the first-member by at least an area of γ·A_FoV_. Thus, when a sensor enters in duty, he will monitor an area equal to γ·A_FoV_ overlapped with the first-member and an area equal to (1 – γ)·A_FoV_ that in the worst case does not overlap with any other member of the cluster. Sensing the whole cluster area with T_interval_ equal to T would result in that an area equivalent to (1 – γ)·A_FoV_ would be monitored every C_size_·T, a value that can be very high. However, using Equation (12), monitoring of the area equivalent to (1 – γ)·A_FoV_ is guaranteed by a monitoring interval that is not superior to T/(1 – γ), that is much lower than C_size_·T.

Sleep/wake up protocols has extensively been studied in the area of wireless sensor networks, mainly for the radio subsystem, [23]. Our clustering algorithm works on the sensing subsystem. It is important to notice that executing object detection does not imply sending packets to the sink. Thus, the sleep/wake up algorithm can be decoupled with the radio subsystem. Sleep/wake up can be based on periodic duty-cycle synchronized by the first-member: every T_p_ period, the sensing subsystem wakes up and performs object detection. However, clock drifts can cause cluster de-synchronization. To handle resynchronization, the system makes use of the beaconing scheme for cluster maintenance: nodes receive periodical beacons from the first-member and vice versa in order to detect new members or to detect members that have died. Beaconing duty-cycling belongs to the radio subsystem and it is independent of the sensing subsystem. That means that waking up the sensor to send a beacon is independent of waking up the sensor to take a picture and perform object detection. Thus, the cluster-head may resynchronize cluster members without need of waking up the sensing subsystem. In case the cluster-head dies, cluster members are reassigned to another clusters or form new cluster/s as explained in Section 3.2.

### 5.2. Power Conservation Evaluation

To evaluate the proposed scheduling scheme in terms of power conservation, we compare the cooperative scheduled scheme with a single-tier network or a tier of a multi-tier architecture consisting of N nodes performing object detection without coordination among them as [14,15,18], in which, nodes are awakened with a time period of T. We note that the evaluation is over the sensing subsystem and that the radio subsystem (*i.e.*, transmission and reception of packets) is not taken into account.

The energy consumed in the network for object detection by N nodes during a duty-cycle interval of T in the non-collaborative scheduling is:

(14) $$E=N\cdot \left(T_{\text{sleep}}\cdot P_{\text{sleep}}+E_{w\_\text{up}}+E_{\text{cap}}+E_{\text{detect}}\right)$$

where T_sleep_ and P_sleep_ are the period and power consumption for a node in sleep mode. E_w_up_, E_cap_ and E_detect_ respectively are the energies consumed in waking up a node, capturing a picture and performing object detection.

**Figure 11.**
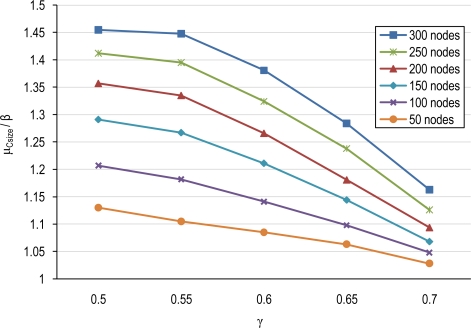
The average of coefficient μ_Csize_/β for several node densities and overlapping scales, γ.

Let us now consider the cooperative scheduling algorithm in a clustered tier/network. Both, the interval between waking up consecutive nodes in the same cluster and the period of waking up a given node are functions of the cluster-size of the cluster which the nodes belong to. In one hand, in clusters with high cluster-size, T_interval_ is small and thus cluster duty-cycle frequency is increased. On the other hand, higher number of nodes in the cluster causes longer periods T_P_ for awaking a given node of the cluster and thus yields an enhancement for power conservation in cluster’s members. Assuming average cluster-size for all clusters in the tier/network, T_P_ will be:

(15) $$T_{P}=\frac{T\cdot \mu_{C_{\text{size}}}}{\mu_{C_{\text{size}}}-\gamma \cdot \left(\mu_{C_{\text{size}}}-1\right)}$$

where T is the base period for waking nodes in the base un-coordinated tier. Figure 11 shows the evolution of T_p_ normalized by T (*i.e.*, μ_Csize_/β) for several node densities and overlapping scales, γ. We may observe that the node average duty-cycle frequency is reduced by factors that are, for example, on the order of 0.78 for a 200 node network and a scale factor of γ = 0.6.

Consequently, the total amount of averaged consumed energy by nodes for object detection in the coordinated tier during T_P_ will be:

(16) $$E_{p}=E+N\cdot P_{\text{sleep}}\cdot \left(T_{p}-T\right)$$

From Equations (15) and (16) we have:

$$E_{P}=E+\frac{\gamma \cdot T\cdot \left(\mu_{C_{\mathrm{size}}}-1\right)}{\mu_{C_{\mathrm{size}}}-\gamma \cdot \left(\mu_{C_{\mathrm{size}}}-1\right)}\cdot N\cdot P_{\mathrm{sleep}}$$

So:

$$\begin{array}{c} \frac{E_{P}}{T_{P}}=\frac{E\cdot \left(\mu_{C_{\text{size}}}-\gamma \cdot \left(\mu_{C_{\text{size}}}-1\right)\right)}{T\cdot \mu_{C_{\text{size}}}}+\frac{\gamma \cdot \left(\mu_{C_{\text{size}}}-1\right)\cdot N\cdot P_{\text{sleep}}}{\mu_{C_{\text{size}}}}\\ \frac{E_{P}}{T_{P}}=\left(1-\frac{\mu_{C_{\text{size}}}-1}{\mu_{C_{\text{size}}}}\cdot \gamma \right)\cdot \frac{E}{T}+\frac{N\cdot \gamma \cdot \left(\mu_{C_{\text{size}}}-1\right)}{\mu_{C_{\text{size}}}}\cdot P_{\text{sleep}}\;\text{where}\;(0<\gamma <1)\;\;\;\text{and}\;\;\;\left(\mu_{C_{\text{size}}}>1\right) \end{array}$$

Therefore, the consumed power is:

(17) $$P_{P}=\lambda \cdot P+\sigma \cdot P_{\text{sleep}}$$

where:

$$\begin{array}{c} \lambda =(1-\frac{\mu_{C_{\text{size}}}-1}{\mu_{C_{\text{size}}}}\cdot \gamma )\;,0<\lambda <1\\ \sigma =\frac{N\cdot \gamma \cdot (\mu_{C_{\text{size}}}-1)}{\mu_{C_{\text{size}}}}\;,0<\sigma <\gamma \cdot N \end{array}$$

Parameter P in Equation (17) is the power consumed in the network with the base un-coordinated mechanism. The consumed power in our scheme (P_P_) is reduced by a factor λ with respect to P. The λ factor depends on the average cluster-size and the overlapping scale factor. As can be observed from Equation (17) increasing μ_Csize_ produces lower values of λ, and thus a saving in energy with respect the uncoordinated system. For example a μ_Csize_ = 1.5 (100 nodes with γ = 0.5) produces a λ = 1 – γ/3 = 0.83 while a μ_Csize_ = 2.15 (200 nodes with γ = 0.5) produces a λ = 1 – 0.53·γ = 0.73. The other term (σ·P_sleep_) in Equation (17) is due to the fact of taking nodes to sleep mode in intervals of duration (T_P_ > T) and then nodes sleep T_p_–T more time than in the un-clustered scheme.

**Figure 12.**
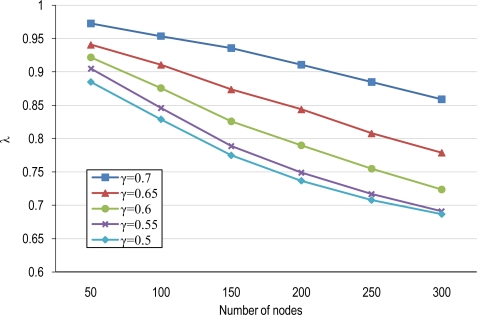
Factor λ in cooperative scheduling for several overlapping scales.

Figure 12 illustrates the impact of factor λ in Equation (17) in terms of node densities for several overlapping scales. From this figure we can see that in high node density tiers, the factor λ is more beneficial since μ_Csize_ is higher and thus there is more potential of cooperation among nodes.

**Figure 13.**
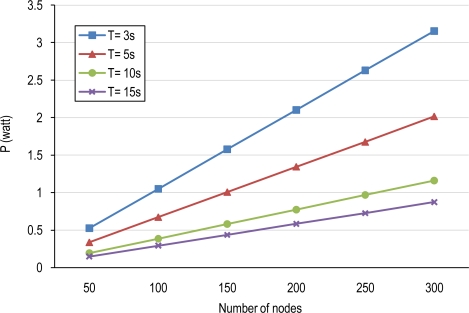
Consumed power (P) for a non-cooperative tier/network of nodes consisting of Cyclops.

Figure 13 shows the consumed power (P) in the base un-coordinated tier for object detection in four cases of period of duty-cycle for different node densities. The consumed power has been computed for nodes consisting of Cyclops as camera sensor embedded in the host MICA II, similar to the tier 1 in [15].

**Figure 14.**
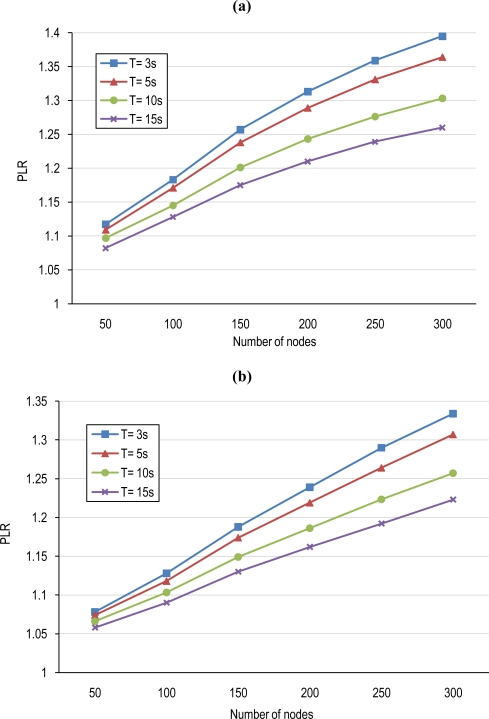
Prolongation Lifetime Ratio (PLR) for different node densities in the clustered tier with an overlapping scale equal to (a) 0.5. (b) 0.6, in four states of base awakening period.

For instance, in the case without coordination, the power consumed in a tier consisting of 200 nodes that performs object detection with a duty cycle of T = 5 s, is 1.344 watts. In the coordinated network with the same number of nodes and an overlapping scale of 0.5, the power consumed by the network would be reduced by a factor λ of 0.737 (see Figure 12) at the cost of increasing 52.60 mW, (σ·P_sleep_). This means a tier power consumption of 1.344 × 0.737 + 0.0526 = 1.043 Watts implying a reduction of 22.39%. Thus, in this case, the Prolongation Lifetime Ratio (PLR) would be of 1.344/1.043 = 1.289. Figure 14a,b shows the prolongation lifetime ratio assuming an overlapping scale of 0.5 and 0.6 for different node densities in four cases of duty-cycle (T). Tiers with high number of nodes have higher capability for cooperation and thus their nodes can conserve considerable amount of energy comparing to sparse networks and consequently, have longer prolonged lifetime. The figure indicates the more prolongation lifetime for dense tiers.

**Figure 15.**
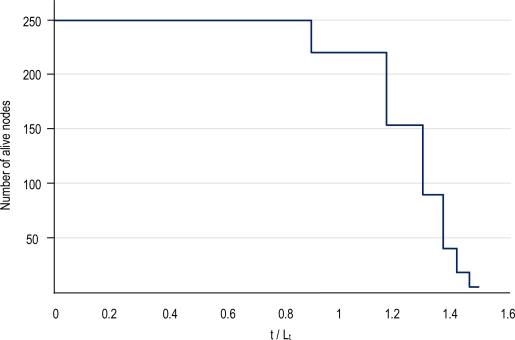
Lifetime in a deployment of 250 nodes.

Finally, the lifetime increment in a deployment of 250 nodes is shown in Figure 15. The lifetime is normalized to L_t_, the time to which all nodes would deplete their batteries without coordination. This lifetime only is due to sensing and thus forwarding and other tasks are not taken into account. We assume that all nodes under an uncoordinated duty-cycle scheme will die at the same time with a difference of T seconds among them (*i.e.*, L_t_ ± T). Note, that if we include the radio subsystem, the lifetime of every node would be less than L_t_. Moreover, L_t_ will depend on the initial energy stored in the nodes. As can be observed, clustering prolongs the lifetime of the network. Single nodes (clusters having only one node) are the first set of dying nodes (*i.e.*, dying at L_t_). Then, cluster members of clusters of size two nodes die at 1.3·Lt and clusters of size three die at 1.43·L_t_. We may observe, thus, increments of 30% and 43% in the lifetime of the sensors for these cluster sizes.

## 6. Conclusions

In this paper, a clustering method for wireless multimedia sensor networks is proposed. Cluster-membership is decided based on FoV overlapping areas instead of radio neighbourhood in contrast to scalar sensors. The main objective of the clustering method is to achieve the ability of cooperation among cluster nodes in sensing and processing applications and thus conserve energy in the network. Coordination in multimedia nodes can considerably prevent wasting of power avoiding redundant sensing, processing or sending similar multimedia data. Therefore, it prolongs network lifetime particularly in networks with a high density of usual low power, low resolution and inexpensive nodes.

A cooperative scheduling method for object detection based on the proposed clustering approach is presented as an example of cluster-based cooperative application. In this scheme, in each cluster, members are scheduled to sequentially wake up, capture an image and monitor the presence of objects in an intermittent manner with an interval depending on the cluster-size and scale of overlapping. Results show how the proposed scheduling method reduces energy consumption of the network, with respect to energy consumption in ordinary un-clustered object detection applications and thus increase the network lifetime especially in dense networks.

## Figures and Tables

**Table 1.** Cluster formation.

**The Algorithm**
1: k = 1, i = 1 // k is index of clusters, i is index of sensors //
2: C_S = {0,...0}, F_S = {0,...,0) // Mask vectors of size N indicating whether node S_i_ is clustered and whether node S_i_ is a first-member //
3: **Repeat:**
4: Create an empty cluster (C_k_): C_k_ = Ø
5: Set C_S_i_ as a clustered multimedia node, member of C_k_
6: Set F_S_i_ as the first member of the cluster C_k_
7: **For all** C_S_j_// un-clustered multimedia nodes //
8: **If** (C_S_j_ = 0)
9: Find intersection polygon of FoVs of S_i_, S_j_
10: Compute D_ij_ //overlapped area between S_i_, S_j_ //
11: **If** (D_ij_ ≥ γ.A_FoV_)
12: Set C_S_j_ as a clustered multimedia node
13: Set C_S_j_ as a member of C_k_
14: **End-If**
15: **End-If**
16: **End-For**
17: i ← next i //next un-clustered node//
18: K = K + 1
19: **Until** (all nodes are clustered)

**Table 2.** Cluster-based Cooperative Scheduling for Object Detection.

**The Algorithm**
1: **For all** C_j_ // all clusters in parallel //
2: i = 0 // start with the first member of each cluster //
3: Wake up member number i
4: Capture an image and then call object detection
5: **If** (detection==true)
6: Send the image to sink
7: **End-If**
8: Delay (T_interval_) // each cluster has a proprietary T_interval_ //
9: i = i + 1(mod C_size_) // select next node of the cluster //
10: **Goto 3**
11: **End-For**
